# Zinc – a scoping review for Nordic Nutrition Recommendations 2023

**DOI:** 10.29219/fnr.v67.10368

**Published:** 2023-12-06

**Authors:** Tor A. Strand, Maria Mathisen

**Affiliations:** 1 Department of Global Public Health and Primary Care, University of Bergen, Bergen, Norway; 2 Innlandet Hospital Trust, Lillehammer, Norway; 3 Department of Medical Microbiology, Drammen Hospital, Drammen, Norway

**Keywords:** zinc, trace elements, cofactors, nutrition recommendations

## Abstract

Zinc is essential for several biological processes including those critical for the immune system, DNA synthesis, cell division, and growth. Zinc is involved in the pathophysiology of chronic diseases and protects proteins and lipids from oxidative damage. Inadequate zinc intake and low plasma zinc concentration are associated to an increased risk of chronic diseases such as cardiovascular diseases and type 2 diabetes; however, the evidence is limited. Zinc deficiency increases the risk of infections and poor growth and may contribute to the high burden of infectious diseases and stunting in children living in low- and middle-income countries. The risk of zinc deficiency in the populations of the Nordic and Baltic countries is low.

## Popular scientific summary

- Zinc has a wide range of crucial physiological functions and is present in every cell in the human body.
- No reliable biomarker of zinc status has been established.
- Severe zinc deficiency increases susceptibility to infections, poor growth, poor wound healing, hair loss, anorexia, altered taste, skin lesions, and eczema.
- The consequences of mild or moderate zinc deficiency are unclear.
- Meat, dairy products, legumes, eggs, fish, and cereals are good food sources of zinc.
- Bioavailability of zinc is affected by the intake of phytate and calcium.

Zinc is essential for DNA synthesis, cell division, and the normal functioning of the immune system (1). Zinc is an essential part of many enzymes involved in the synthesis, metabolism, and turnover of proteins, carbohydrates, lipids, nucleic acids, and vitamins. Zinc-containing enzymes include superoxide dismutase, alkaline phosphatase, and alcohol dehydrogenase. Zinc has also been related to maintaining adequate bone density, cognitive function, fertility and reproduction, metabolism of fatty acids, acid-base metabolism, vitamin A metabolism, and vision (1, 2). The consequences of severe zinc deficiency are increased susceptibility to infections, poor growth, poor wound healing, hair loss, anorexia, altered taste, skin lesions, and eczema. Many children in low- and middle-income countries (LMIC) have inadequate zinc intake that impairs their immune system and growth. Large randomized controlled trials (RCTs) have shown that routine zinc supplementation to apparently healthy children from LMICs reduces the incidence of diarrhoea, the risk of acquiring severe illness, and the risk of acute lower respiratory infections (3). Evidence from RCTs has also demonstrated that oral zinc reduces the incidence and duration of common colds (4). This scoping review aims to describe the totality of evidence for the role of zinc for health-related outcomes as a basis for setting and updating dietary reference values (DRVs) for the Nordic Nutrition Recommendations (NNR) 2023 (Box 1).

Box 1 Background papers for Nordic Nutrition Recommendations 2023

- This paper is one of many scoping reviews commissioned as part of the Nordic Nutrition Recommendations 2023 (NNR2023) project (5)
- The papers are included in the extended NNR2023 report, but, for transparency, these scoping reviews are also published in Food & Nutrition Research
- The scoping reviews have been peer reviewed by independent experts in the research field according to the standard procedures of the journal
- The scoping reviews have also been subjected to public consultations (see the report to be published by the NNR2023 project)
- The NNR2023 committee has served as the editorial board
- While these papers are the main fundament, the NNR2023 committee has the sole responsibility for setting dietary reference values in the NNR2023 project.

## Methods

This review follows the protocol developed within the NNR2023 project (5). The sources of evidence used in the scoping review follow the eligibility criteria described previously (6). The main literature search for this scoping review was performed on October 23rd, 2019 in MEDLINE with a search string: zinc[MeSH Terms] AND (“2011”[Date – Publication] : “3000”[Date – Publication]) AND review[Publication Type] AND Humans[Filter] AND (“Diet” OR “Dietary” OR “Food” OR “Nutrition” OR “Nutritional”). The number of hits was 385. Based on the title, 92 articles were identified of which 29 were considered relevant based on the full articles. Of these 29, only 5 were systematic reviews; however, no strong evidence was identified that would cause any changes to the DRVs. Zinc was not selected for a *de novo* systematic review and no qualified systematic review on zinc was identified by the NNR project (7).

We have also identified other reviews that were relevant for the section describing the health outcomes through a ‘snowballing approach’ (i.e. by tracking down citations in the selected reviews).

## Physiology

Zinc is a widespread element that exists as a stable divalent cation (Zn^2+^). It has a wide range of vital physiological functions and is present in every cell in the human body (1). Zinc has a structural and catalytic role in each of the seven classes of enzymes (1). It is involved in the synthesis, metabolism, and turnover of proteins, carbohydrates, lipids, nucleic acids, and some vitamins. An essential structural role of zinc is zinc motifs (zinc fingers) for transcription factors. Such zinc finger proteins represent 8% of the human genome and account for a significant part of the zinc requirement (8, 9). The functions of zinc finger proteins include controlling and modifying gene transcription and translation and signal transduction. Other regulatory functions include regulation of gene expression and intracellular signalling, especially through regulation of kinase and phosphorylase activity (8).

Zinc also acts as a cofactor for several key enzymes important for reducing oxidative stress and zinc induces the synthesis of metallothioneins, which are effective in reducing free radicals (1).

Absorption of zinc is dose dependent and occurs via enterocytes, mainly in the upper part of the small intestine (10). The luminal content of phytate and calcium negatively impacts the amount of zinc available for absorption. Diets dominated by plant foods are rich in phytate, and vegetarians may accordingly be at risk of low zinc absorption (11, 12).

Absorbed zinc is transported in the blood, mostly bound to albumin (1). Most of the zinc in the body (2.5 g in men and 1.5 g in women) is located in muscle tissue and bones, and only 0.1% is found in plasma (1). The liver acts as a short-term store where zinc can be mobilized to plasma when needed. There are no significant long-term stores for zinc in the human body. In times of depletion, bone zinc may be released into the circulation, albeit at a much slower rate than what can be released from the liver (1).

Zinc is excreted mainly through the through the faeces, while the kidneys and skin are minor excretion routes. Strong homeostatic mechanisms keep the zinc content of tissues and fluids constant over a wide range of intakes through changes in excretion and absorption. During lactation, the content of zinc in breast milk declines and is particularly low after 6 months (13).

## Assessment of zinc status

The biomarker recommended to assess zinc status is serum or plasma zinc concentration (14). Due to substantial intra-individual variability in the plasma zinc concentration, this biomarker should be used with caution and is best suited to estimate population status. Serum zinc concentrations fall sharply when dietary zinc intakes are less than ~2 to 3 mg/day but rise slightly but continuously when intakes are greater, reaching a plateau when intakes reach ~25 to 30 mg/day (15). However, because the plasma zinc concentration is also influenced by factors unrelated to zinc status, such as food intake, inflammation, and tissue anabolism or catabolism, the measurement cannot be used for estimating zinc status. Other markers based on enzyme activities are also available but are not in routine clinical use or commonly used in population-based studies (8).

### Dietary intake in Nordic and Baltic countries

Meat, dairy products, legumes, eggs, fish, grains, and grain-based products are rich dietary zinc sources (16). According to the NNR review on adult nutrient intake in the Nordic and Baltic countries, the mean intake from all countries was above the recommended intake (RI) set in NNR2012 (17). Zinc intake in the Norwegian population was, however, not included in this report. In 2017, the Norwegian Scientific Committee for Food and Environment (VKM) estimated the intake of zinc from the diet, including fortified products, in all age groups of the population above 1 year of age (18). This report found the same intake levels in Norway as in the Baltic and other Nordic countries. The VKM report also included data on the distribution of intakes and found that the risk of having deficient or excess zinc intake in the Norwegian population was low.

## Health outcomes relevant for Nordic and Baltic countries

### Deficiencies

Well-defined clinical zinc deficiency has only been reported in a limited number of cases that are related to incomplete total parenteral nutrition, malabsorption, rare inherited diseases, and the use of drugs (19). Estimates based on evaluation of zinc intakes and diet composition in different parts of the world suggest that the populations of many countries in Asia and Africa are at high risk for developing zinc deficiency and that the risk is low in European countries and North America (2). Some of the clinical manifestations of severe zinc deficiency are growth retardation, delayed sexual maturation, skin lesions adjacent to the body orifices, hair loss, as well as behavioural disturbances (19). These clinical signs have almost exclusively been observed in subjects with the inborn error in zinc transport (acrodermatitis enteropathica) and adolescents subsisting on diets with presumably very low zinc availability (1). The consequences of moderate and mild zinc deficiency remain unclear. Zinc supplements, even at or slightly above the recommended daily intake (RDI), can cause nausea and vomiting (1, 20). Zinc intake can also reduce the absorption of other divalent cations such as copper (Cu), iron (Fe), and calcium (Ca) (1, 20). Impaired absorption of these minerals might theoretically cause impaired immunity, anaemia, and poor bone mineralization, but such effects of supplementing with zinc have not been shown.

### Zinc toxicity

Although it may induce vomiting (21, 22), zinc is not considered to be toxic even in relatively high doses. Excess zinc in the diet is not absorbed and stored in the body for later use, and there is no known disorder that is associated with its accumulation (1). The effect of zinc supplementation on plasma Cu concentration has also been used to generate the upper level of intake (UL) for zinc. In 2006, the European Food Safety Authority (EFSA) set an UL for zinc of 25 mg/day for adults (23).

### Chronic diseases in adults

The potential roles of zinc in preventing non-communicable diseases have been of interest (24). Zinc plays a crucial role in insulin action in peripheral tissue and may improve insulin sensitivity (25). Cell and animal studies have shown the protective effects of zinc against risk factors of cardiovascular diseases (CVD), such as the development of atherosclerosis (24). However, there is limited evidence for beneficial effects of zinc in preventing CVD and type 2 diabetes in the general population. There is, however, some evidence that improving zinc status may have a role in the secondary prevention of these conditions (26).

### Growth and development

Zinc is important for growth and normal development in early life, and poor growth is a key sign of zinc deficiency in all mammals (1). Stunting and frequent infections seen in many marginalized populations have therefore been linked to inadequate zinc intake, recognizing the possible public health importance of zinc deficiency (27). For the last 30 years, several RCTs on the effect of zinc to improve growth have been undertaken (2, 16, 21, 28). Results from the most recent meta-analyses indicate that the relative contribution of dietary zinc in childhood stunting is limited (29).

### Infectious diseases

Zinc is crucial for the normal functioning of the immune system, and zinc deficiency affects both innate and adaptive immunity (30). Inadequate zinc status is associated with an increased risk of infections, and several zinc supplementation trials have shown that zinc reduces the risk of childhood diarrhoea and pneumonia (3, 4). Zinc supplementation can also be used to prevent upper respiratory infections in adults; oral zinc administered within 24 hours of the onset of symptoms has been shown to reduce the duration and severity of common colds in otherwise healthy people (4). In these supplementation trials, zinc was provided in doses up to 150 mg/day for up to a few months. Zinc is recommended by the World Health Organization for treating acute diarrhoea in children in LMICs. The role of zinc in infections in a Nordic and Baltic setting is uncertain.

### Pregnancy

Zinc is also important in pregnancy and for the developing foetus. A Cochrane systematic review of 21 RCTs concluded that zinc supplementation could reduce the risk of preterm birth. However, the studies included in this review were undertaken in LMIC settings that do not reflect the zinc status in the Nordic and Baltic countries (31).

## Requirement and recommended intakes

### Adults

In NNR2012, the average dietary zinc requirements (AR) were estimated based on daily losses through the kidneys, skin, semen, or menses and the gastrointestinal tract (faeces). The dietary requirement is also dependent on the fraction of zinc absorbed from the diet. Fractional zinc absorption is dependent on zinc content; when intakes are increased, the proportion absorbed decreases. The composition of other nutrients in the diet will also affect the fraction of zinc absorbed. At low intakes of zinc in diets with no inhibitors, the fractional absorption can be >50%, but the fraction can be as low as 10% (32). In NNR2012, the fractional absorption from a mixed animal and vegetable protein diet more realistic for Nordic and Baltic conditions was assumed to be 40%. The estimated endogenous losses were 2.67 mg/day for men and 2.40 mg/day for women. With an inter-individual requirement variability of 15%, the RI was set to 7 mg/day for women and 9 mg/day for men.

It is possible that a more plant-based diet with a higher content of chelating substances such as phytic acid and tannins may increase zinc requirements. Furthermore, since animal-source foods are the main sources of zinc, a shift to a predominant plant-based diet might result in lower mean intakes and a higher proportion of the population that falls below the AR. It should be noted, however, that the risk of deficiency in the Nordic and Baltic countries is low. In 2014, EFSA updated their DRVs for zinc and provided adjusted population reference intakes (PRI) according to the intake of phytic acid (16). In this scientific opinion report, they arrived at PRIs that were close to the RIs used in NNR2012.

### Children

There is insufficient data on the endogenous losses of zinc at different intakes for children. The zinc requirements for adequate growth are approximately 175 µg/kg/day during the first month and then approximately 30 µg/kg/day for the next 9–12 months (33). For growing children, the need for zinc is based on basal losses of 0.1 mg/kg and zinc content in new tissue of 30 µg/kg. For adolescent growth, the estimated average zinc content in new tissue is 23 µg/kg. Therefore, the physiological requirements for rapidly growing adolescents can be increased by 0.3–0.4 mg/day. Applying the same principles as for adults, the recommended daily zinc intake varies from 2 mg in the youngest age group to 12 mg for adolescent boys.

### Pregnancy and lactation

The total need for zinc during pregnancy for the foetus, placenta, and other tissues is approximately 100 mg (34). This additional need for zinc in pregnancy can be met by increasing zinc intake or adjusting zinc homeostasis. There is no evidence that pregnant women increase their intake of zinc, so homeostatic adjustments in zinc utilization are likely the primary mechanism for meeting the additional zinc demands for reproduction (34). It is assumed that increased efficiency of zinc absorption or other metabolic changes occurs during pregnancy ensuring that the requirement for zinc can be met with an unchanged intake. However, studies on zinc metabolism during pregnancy are inconclusive (35, 36). In NNR2012, the RIs were based on an estimated increase in the physiological requirement by 0.7 mg/day with adjustment for absorption. The additional dietary intake in pregnancy was set to 2 mg/day.

The zinc content in breast milk is approximately 2.5 mg/L in the first month of lactation and decreases to about 0.7 mg/L after 4 months (33). This means that the zinc requirement of lactating women is higher than non-lactating women. A fractional increase in zinc absorption of up to 70–80% has been shown for lactating women compared with non-lactating postpartum or never-pregnant women (36, 37). Release of zinc from bone tissue could also explain why zinc concentrations in breast milk are relatively independent of the mother’s zinc intake and do not seem to result in zinc deficiency of the mother even after a long period of lactation. An elevated intake corresponding to the zinc content in breast milk is recommended for women lactating for a long time, that is, a physiological need of 1.7 mg/day. With adjustment for absorption, the additional dietary intake of about 4 mg/day should be considered.

### Data gaps for future research

The DRVs for zinc vary between countries and regions, which reflects the uncertainties in the DRV estimates. This variability is, in part, due to the variability in fractional absorption and uncertainty related to the health consequences of mild to moderate zinc deficiency. The consequences of mild or moderate zinc deficiency and the identification of reliable biomarkers for zinc status are important knowledge gaps. Furthermore, it is expected that the intake of animal-source foods will decrease, and how this will influence zinc status and the risk for zinc deficiency in the Nordic and Baltic countries is an important research question.
